# Development and validation of mPCR-CEFA for detecting multiple deletion and non-deletion thalassemia genotypes

**DOI:** 10.3389/fgene.2025.1564565

**Published:** 2025-07-08

**Authors:** Jingping Xu, Baoyan Ren, Qixun Fang, Kangfeng Lin, Xingan Xing, Jingting Lin

**Affiliations:** ^1^ School of Chemistry and Chemical Engineering, South China University of Technology, Guangzhou, China; ^2^ Yaneng BIOscience (Shenzhen) Co., Ltd., Shenzhen, China; ^3^ Photonics Research Centre, Shenzhen University, Shenzhen, China

**Keywords:** thalassemia, multiplex PCR, capillary electrophoresis, genotyping, genetic screening, prenatal diagnosis

## Abstract

**Background:**

Thalassemia is a common hereditary blood disorder caused by genetic variants in globin genes, leading to abnormal hemoglobin production. Rapid and accurate genotyping is essential for molecular screening and prenatal genetic diagnosis to prevent the birth of individuals with severe forms of the disease.

**Methods:**

We developed a multiplex PCR-capillary electrophoresis fragment analysis (mPCR-CEFA) method to detect 16 α-thalassemia and 24 β-thalassemia genotypes simultaneously. Genomic DNA extracted from clinical blood samples underwent a two-tube multiplex PCR amplification. The amplification products were analyzed using capillary electrophoresis to detect mutation peaks in different fluorescent channels and to calculate α1/α2 and Y1/Y2 ratios for genotype determination. The performance of mPCR-CEFA was validated against conventional methods, including Gap-PCR and PCR-RDB.

**Results:**

The α1/α2 and Y1/Y2 peak ratios exhibited stable and reproducible values, allowing for precise genotyping of thalassemia events involving homologous recombination, such as -α^3.7^, -α^4.2^, ααα^anti3.7^, ααα^anti4.2^ and HKαα. Mutation peaks in different fluorescent channels also facilitated the differentiation of various genotypes, including deletion and non-deletion types. The method demonstrated a high accuracy rate of 99.5%. It successfully detected complex compound genotypes like α^CD 74^α/−α^4.2^, β^CD 17^/β^N^ and α^WS^α/--^SEA^, β^CD 37^/β^N^ (or α^WS^α/--^SEA^, β^CD 37^/β^CD 37^), which were challenging for traditional approaches.

**Conclusion:**

The mPCR-CEFA method is a reliable, efficient, and scalable tool for genetic diagnosis of thalassemia. Its ability to detect multiple genotypes simultaneously and resolve complex cases makes it particularly valuable for large-scale screening and clinical applications. This approach holds significant potential for improving thalassemia prevention strategies and supporting public health efforts in high-prevalence regions.

## 1 Introduction

Thalassemia is a prevalent genetic disorder of the blood system, resulting from variants in globin genes, which lead to abnormal hemoglobin synthesis and hereditary hemolytic diseases (Kattamis et al., 2022). Globally, there are approximately 345 million thalassemia gene carriers, including about 30 million in China. The number of major and intermediate patients is estimated to be about 300,000, increasing by approximately 10% annually (Wang et al., 2023). The prevalence of thalassemia is notably high in regions located south of the Yangtze River in China, especially in Guangxi, Guangdong, and Hainan, which brings a heavy burden to families and social medical care (Lai et al., 2017).

Based on the different types of globin, thalassemia can be classified into α-, β-, γ-, and δ-thalassemia, of which α- and β-thalassemia are the most common. According to the severity of clinical symptoms, thalassemia can be divided into silent carrier state, minor, intermedia, and major. Silent or mild thalassemia presents with no obvious or mild symptoms that do not significantly impact daily life and work. However, severe thalassemia may manifest shortly after birth with symptoms such as hepatosplenomegaly, jaundice, anemia, and stunted growth, and can even be life-threatening (Lou et al., 2023). Currently, there is no radical cure for thalassemia with the treatments available. Genetic counseling, pre-marital examinations, and prenatal screenings are essential in controlling the spread of thalassemia. Reducing or preventing the birth of individuals with severe forms of thalassemia remains the most effective preventive measure (Modell and Darlison, 2008; Long et al., 2024).

Laboratory testing of thalassemia mainly includes two methods: screening and genetic diagnosis. The screening method can be further categorized into routine blood tests and hemoglobin tests. However, missed diagnoses are common due to the lack of typical clinical characteristics in partially silent, mild, or compound thalassemia (Esmaeilzadeh et al., 2022; Xu et al., 2022; Yang et al., 2022). Genetic testing can greatly improve the accuracy of thalassemia detection and reduce the miss rate of thalassemia, and it is regarded as the gold standard for thalassemia diagnosis (Munkongdee et al., 2020; Luo et al., 2022b). Commonly used genetic methods include Gap-PCR and PCR-reverse dot blot (PCR-RDB) (Jin et al., 2024), which are primarily employed to detect four deletional α-thalassemia, three non-deletional α-thalassemia, and seventeen β-thalassemia variants. Other molecular diagnostic techniques comprise amplification refractory mutation system PCR (ARMS-PCR) (Baig, 2007), multiplex ligation-dependent probe amplification (MLPA) (Jomoui et al., 2023), qPCR (Zhou et al., 2013; Long, 2016), fluorescence PCR probe melting curve analysis (PMCA) (Huang et al., 2016), gene microarrays (Pornprasert et al., 2019), and DNA sequencing (Wu et al., 2022; Liang et al., 2023). Although these methods offer high accuracy, each has certain limitations in detection throughput, operational complexity, and cost-effectiveness. Therefore, there is a need to develop new technologies that can overcome these deficiencies.

Multiplex PCR-capillary electrophoresis fragment analysis (mPCR-CEFA) is an efficient molecular diagnostic technique that integrates multiplex fluorescent PCR with capillary electrophoresis separation, providing high-throughput and parallel detection capability. By using a panel of fluorescently labeled target-specific primers, the method can amplify multiple targets simultaneously in a single PCR reaction. The amplified products are then separated by capillary electrophoresis, and DNA fragments of different sizes are accurately distinguished based on their migration rates and quantitatively analyzed using a fluorescence detection system. Even when the amplicons are present in low amounts or differ in length by only one base pair, mPCR-CEFA enables clear peak resolution and precise identification. Compared with traditional Gap-PCR and PCR-RDB methods, mPCR-CEFA exhibits higher specificity, superior resolution, and greater operational simplicity, making it well-suited for rapid and accurate diagnosis of various thalassemia gene variants (Qin et al., 2003; Zhang et al., 2011).

The present study established a genotyping method based on mPCR-CEFA that can detect sixteen α-thalassemia and twenty-four β-thalassemia variants (Supplementary Table S1). This method distinguishes five genotypes including -α^3.7^, -α^4.2^, ααα^anti3.7^, ααα^anti4.2^, and HKαα through copy number analysis of α1/α2 and Y1/Y2, and identifies the other thirty-five genotypes by detecting characteristic mutation peaks. Two separate PCR reactions were conducted, and their products were pooled into a single well for capillary electrophoresis, enabling simultaneous analysis of up to 40 thalassemia genotypes across 96 samples. This approach notably broadens the detection range and improves the detection efficiency. It is of great significance to carry out molecular screening and prenatal genetic diagnosis within large populations for the effective prevention of thalassemia.

## 2 Materials and methods

### 2.1 Samples

All samples were derived from stored blood samples of thalassemia patients, involving 40 genotypes. Informed consent was obtained from all subjects, and the study was approved by the Medical Ethics Committee of Shenzhen University Medical School. A 2 mL sample of anticoagulated peripheral blood was collected from each patient. Genomic DNA was extracted using the MEB human genome DNA extraction kit (Yaneng BIO, China), with concentration and purity assessed using NanoDrop 2000 (Thermo Fisher Scientific, United States) and stored at −80°C until further use.

### 2.2 Primer design

**FIGURE 1:**
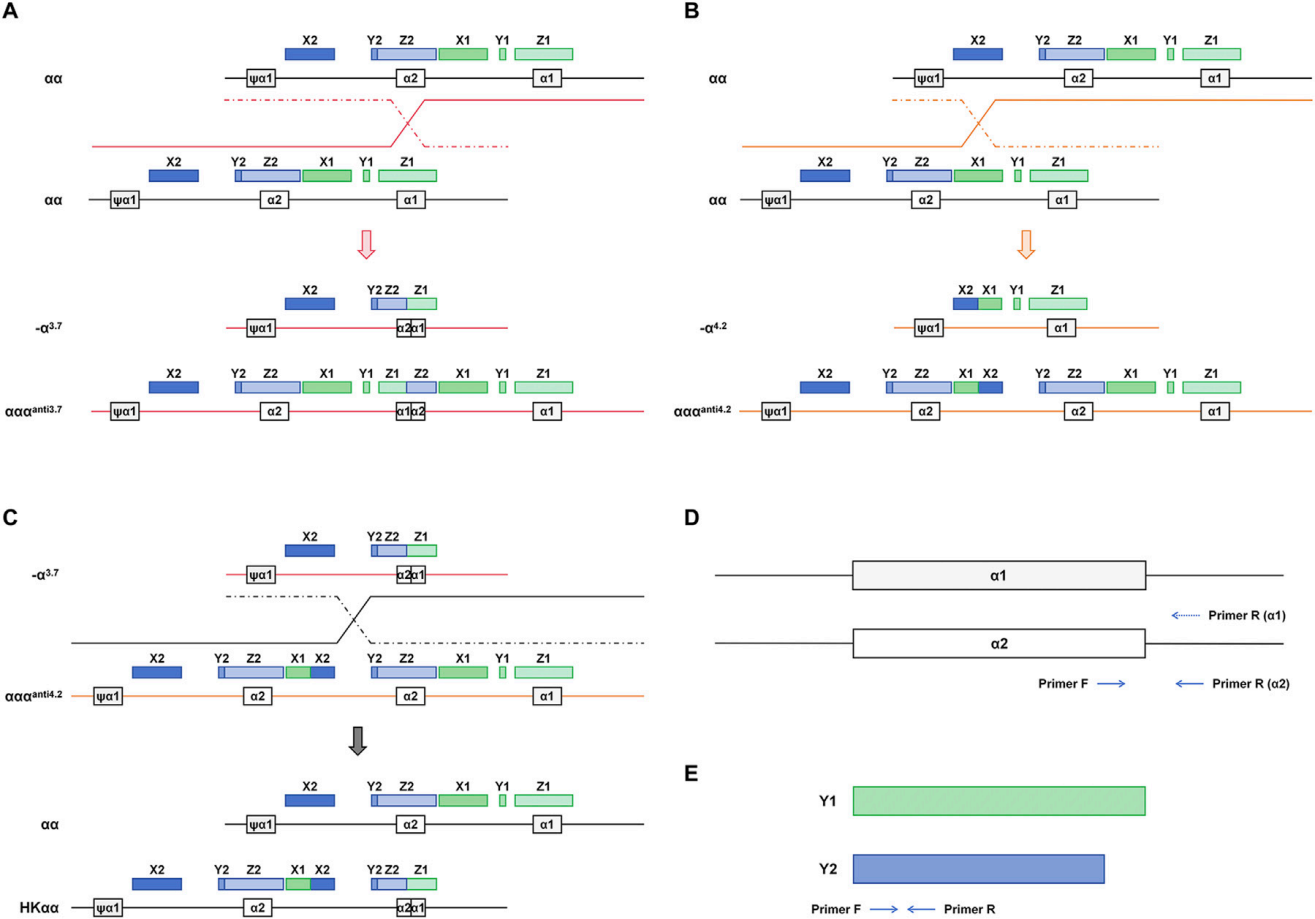
Mechanism of homologous recombination events in α-globin gene and associated thalassemia genotypes. **(A)** crossover between the mispaired Z boxes gives rise to the -α^3.7^ and ααα^anti3.7^ chromosomes. **(B)** crossover between misaligned X boxes gives rise to -α^4.2^ and ααα^anti4.2^. **(C)** crossover occurring in the X2 to Z2 box between -α^3.7^ and ααα^anti4.2^ generates the HKαα allele. **(D)** α1 and α2 amplification primer design strategy. **(E)** Y1 and Y2 amplification primer design strategy.

The primer design referred to the literature previously reported (Long et al., 2013). In addition to the primers targeting the Y1 and Y2 regions, the present study introduced specific primers for the α1 and α2 regions. This approach utilizes the homologous recombination of α-globin gene segments involving X, Y, and Z boxes. These segments undergo unequal crossing-over or recombination, resulting in distinct α-globin gene configurations and genotypes (Higgs et al., 1989; Wang et al., 2005). Specifically, recombination within the Z homologous boxes leads to the deletion of a 3.7 kb DNA fragment on one chromosome (resulting in -α^3.7^), while the other chromosome may exhibit an α triplication (ααα^anti3.7^) (Figure 1A). Similarly, recombination within the X homologous boxes causes the deletion of a 4.2 kb DNA fragment on one chromosome (resulting in -α^4.2^), with the other chromosome potentially forming an α triplication (ααα^anti4.2^) (Figure 1B). The HKαα genotype arises from an unequal exchange between -α^3.7^ and ααα^anti4.2^, encompassing the α2 gene, a fusion gene composed of X1 and X2, and a fusion gene formed by α2 and α1 (Figure 1C). Primers for α1 and α2 were designed on the right side of the gene, enabling the detection of the rightward homologous genes in the presence of α1-α2 fusion genes, with α1 and α2 sharing the upstream primer (Figure 1D). Primers for Y1 and Y2 were designed within the homologous region using a pair of shared forward and reverse primers (Figure 1E).

In addition, primers were designed based on Gap-PCR for deletion-type thalassemia such as --^SEA^, --^THAI^, -α^2.4^, -α^21.9^, -α^27.6^, Chinese ^G^γ^+^(^A^γδβ)^0^, SEA-HPFH and Taiwanese deletion beta-thalassemia, as well as fusion gene. For non-deletion thalassemia alleles including α^CS^α, α^QS^α, α^WS^α, α^CD 30^α, Hb Q-Thailand (α^CD 74^α), β^−90^, β^−50^, β^−29^, β^−28^, β^Cap+40–43^, β^Init CD^, β^CD 5^, β^CD 14/15^, β^CD 17^, β^CD 26^, β^CD 27/28^, β^IVS−I−1^, β^IVS−I−5^, β^CD 37^, β^CD 41/42^, β^CD 43^, β^CD 54–58^, β^CD 71/72^, β^CD 95^, β^IVS−II−5^ and β^IVS−II−654^, the ARMS-PCR method was employed. Primers for detecting α-chain variants were labeled with VIC fluorescence, while those targeting β-chain variants were labeled with FAM fluorescence. To differentiate heterozygous and homozygous forms of certain β non-deletion variants, additional detection primers specific to wild-type alleles at the corresponding variant sites were designed and labeled with NED fluorescence. For internal control, specific primers were designed for the conserved region of GAPDH and gender identification gene AMEL. The primer sequences were as follows: GAPDH-forward: 5′6-FAM-TGGTATCGTGGAAGGACTCATGGTAT-3′; GAPDH-reverse: 5′-GAG​GAG​CCA​GTC​TTG​GAT​GAG​AAA​GG-3′; AMEL-forward: 5′VIC-CCCTGGGCTCTGTAAAGAATAGTG-3′; and AMEL-reverse: 5′-ATC​AGA​GCT​TAA​ACT​GGG​AAG​CTG-3′. Furthermore, to enable simultaneous detection of multiple genotypes, amplicons with the same fluorescence label were designed to be at least 3 bp apart, and all amplicon lengths were controlled within the range of 80–600 bp to facilitate the differentiation of various target fragments by capillary electrophoresis (Supplementary Table S2). All primers were synthesized by Bioligo (China).

### 2.3 mPCR-CEFA

A multiplex PCR assay was used to detect multiple thalassemia genotypes in a two-tube reaction, followed by mixing the amplification products from both tubes in a 1:1 ratio into a single detection tube for capillary electrophoresis fragment analysis. The method involves assessing the product peaks of different fragment lengths at each site in every channel and performing quantitative analysis by calculating the peak height ratios of α1 to α2 and Y1 to Y2, allowing for the simultaneous identification of various thalassemia genotypes. The total volume of each multiplex PCR reaction was 25 μL, including 16 μL multiplex PCR reaction mix (Vazyme, China), 4 μL primer mix, and 5 μL DNA template. The amount of DNA template ranged from 50 to 100 ng. The PCR amplification cycling conditions were as follows: initial denaturing 30 s at 95°C for one cycle, amplification of denaturing 30 s at 95°C, annealing 30 s at 62°C, and extension 30 s at 72°C for 30 cycles, final extension 30 min at 62 °C for one cycle, and cooling to 4 °C. After amplification, 0.5 μL PCR product from each tube was pooled together with 10 μL Hi-Di formamide (Applied Biosystems, United States) and 0.2 μL Liz600 (Applied Biosystems, United States). The mixture was denatured at 95°C for 3 min and transferred to ice for at least 2 min. Then the samples were loaded onto ABI 3500DX and run on a POP7 polymer with a 50-cm capillary. The obtained results were analyzed using GeneMapper IDX (Applied Biosystems, United States).

### 2.4 Genotype validation of samples

Conventional Gap-PCR and PCR-RDB assays were used for comparative analysis. The Thalassemia Gene Detection Kit (PCR-RDB) detects three common α deletions (-α^3.7^, -α^4.2^, and --^SEA^), three α non-deletions (α^CS^α, α^QS^α, and α^WS^α), and seventeen β-chain variants (41–42M, 654M, −28M, 71–72M, 17M, βEM, IVS-I-1M, IVS-I-5M, 27/28M, 43M, −29M, −30M, 31M, −32M, 14–15M, IntM and CAPM). The Deletion Type β-Thalassemia Gene Detection Kit (Gap-PCR) can detect two β deletions (Chinese ^G^γ^+^(^A^γδβ)^0^ and SEA-HPFH). Six α-Thalassemia Gene Detection Kit (PCR-electrophoresis) can detect six types of α-thalassemia (HKαα, --^THAI^, fusion gene, ααα^anti3.7^, ααα^anti4.2^, and −α^27.6^). All kits were sourced from Yaneng BIO (China), and the experimental procedures were carried out according to the manufacturer’s instructions.

### 2.5 Data analysis

Statistical analysis was performed by using GraphPad Prism 8 software, and the data are presented as mean ± standard deviation (SD) in Table 1. The results of the methods were evaluated by calculating their accuracy and concordance rate.

**TABLE 1:** Theoretical and measured ratios of α1/α2 and Y1/Y2 for various thalassemia genotypes.

Genotype	Theoretical ratios		Measured ratios	
	α1:α2	Y1:Y2	α1:α2	Y1:Y2
αα/αα	1:1	1:1	1.04 ± 0.07	0.92 ± 0.07
-α^3.7^/αα	2:1	1:2	2.02 ± 0.14	0.48 ± 0.04
-α^4.2^/αα	2:1	2:1	1.88 ± 0.15	2.05 ± 0.18
ααα^anti3.7^/αα	2:3	3:2	0.74 ± 0.11	1.52 ± 0.09
ααα^anti4.2^/αα	2:3	2:3	0.57 ± 0.10	0.53 ± 0.03
HKαα/αα	1:1	1:3	0.97 ± 0.10	0.35 ± 0.03

## 3 Results

### 3.1 Using α1/α2 and Y1/Y2 to diagnose genotypes of thalassemia formed by homologous recombination

**FIGURE 2:**
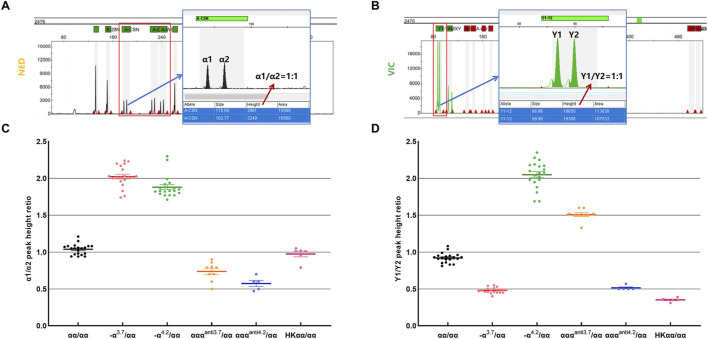
Validation of α1/α2 and Y1/Y2 peak height ratios across thalassemia genotypes. **(A)** Schematic of the capillary electrophoresis profile and peak height calculation for the wild-type α1 and α2. **(B)** Schematic of the capillary electrophoresis profile and peak height calculation for the wild-type Y1 and Y2. **(C)** Statistics of α1/α2 peak height ratio in samples with different genotypes. **(D)** Statistics of Y1/Y2 peak height ratio in samples with different genotypes. n = 20 for αα/αα; n = 20 for -α^3.7^/αα; n = 20 for -α^4.2^/αα; n = 9 for ααα^anti3.7^/αα; n = 5 for ααα^anti4.2^/αα; n = 6 for HKαα/αα.

In this study, we developed a method for genotyping thalassemia variants including -α^3.7^, -α^4.2^, ααα^anti3.7^, ααα^anti4.2^ and HKαα, by leveraging the quantitative capability of mPCR-CEFA to assess the peak height ratios of α1 to α2 and Y1 to Y2. According to the primer design principles described above, the copy numbers of homologous segments α1, α2, Y1, and Y2 vary among different genotypes. In the capillary electrophoresis profiles, the peak height reflects the amount of amplified product, which corresponds to the initial DNA quantity. The α1 and α2 peaks were detected in the NED channel, while the Y1 and Y2 peaks in the VIC channel. Each peak was identified by its amplicon size, and peak height was quantitatively analyzed using GeneMapper IDX. After optimization of the primers, the α1, α2, Y1, and Y2 peaks of different genotypes were successfully and specifically detected. By measuring the peak height ratios of α1 to α2 and Y1 to Y2, the genotype of each sample can be accurately determined (Figures 2A, B).

Theoretical ratios for α1/α2 and Y1/Y2 are as follows: for the wild-type αα/αα, the ratio of α1/α2 is 1:1 and Y1/Y2 is 1:1; for the -α^3.7^/αα genotype, the ratio of α1/α2 is 2:1 and Y1/Y2 is 1:2; for the -α^4.2^/αα genotype, the ratio of α1/α2 is 2:1 and Y1/Y2 is 2:1; for the ααα^anti3.7^/αα genotype, the ratio of α1/α2 is 2:3 and Y1/Y2 is 3:2; for the ααα^anti4.2^/αα genotype, the ratio of α1/α2 is 2:3 and Y1/Y2 is 2:3; and for the HKαα/αα genotype, the ratio of α1/α2 is 1:1 and Y1/Y2 is 1:3 (Table 1). Extensive testing and validation showed that the measured values of α1/α2 and Y1/Y2 for samples were stable and closely matched theoretical values (Table 1; Figures 2C, D). Specifically, in αα/αα samples, the average measured ratio of α1/α2 was 1.04 and Y1/Y2 was 0.92; in -α^3.7^/αα samples, α1/α2 was 2.02 and Y1/Y2 was 0.48; in -α^4.2^/αα samples, α1/α2 was 1.88 and Y1/Y2 was 2.05; in ααα^anti3.7^/αα samples, α1/α2 was 0.74 and Y1/Y2 was 1.52; in ααα^anti4.2^/αα samples, α1/α2 was 0.57 and Y1/Y2 was 0.53; and in HKαα/αα samples, α1/α2 was 0.97 and Y1/Y2 was 0.35. These results demonstrate the quantitative accuracy and robustness of the mPCR-CEFA method in differentiating thalassemia genotypes. Due to varying primer amplification efficiencies across different samples, it is reasonable for the measured values to fluctuate around the theoretical values.

### 3.2 Diagnosing thalassemia genotypes based on mutation peaks in different fluorescent channels

**FIGURE 3:**
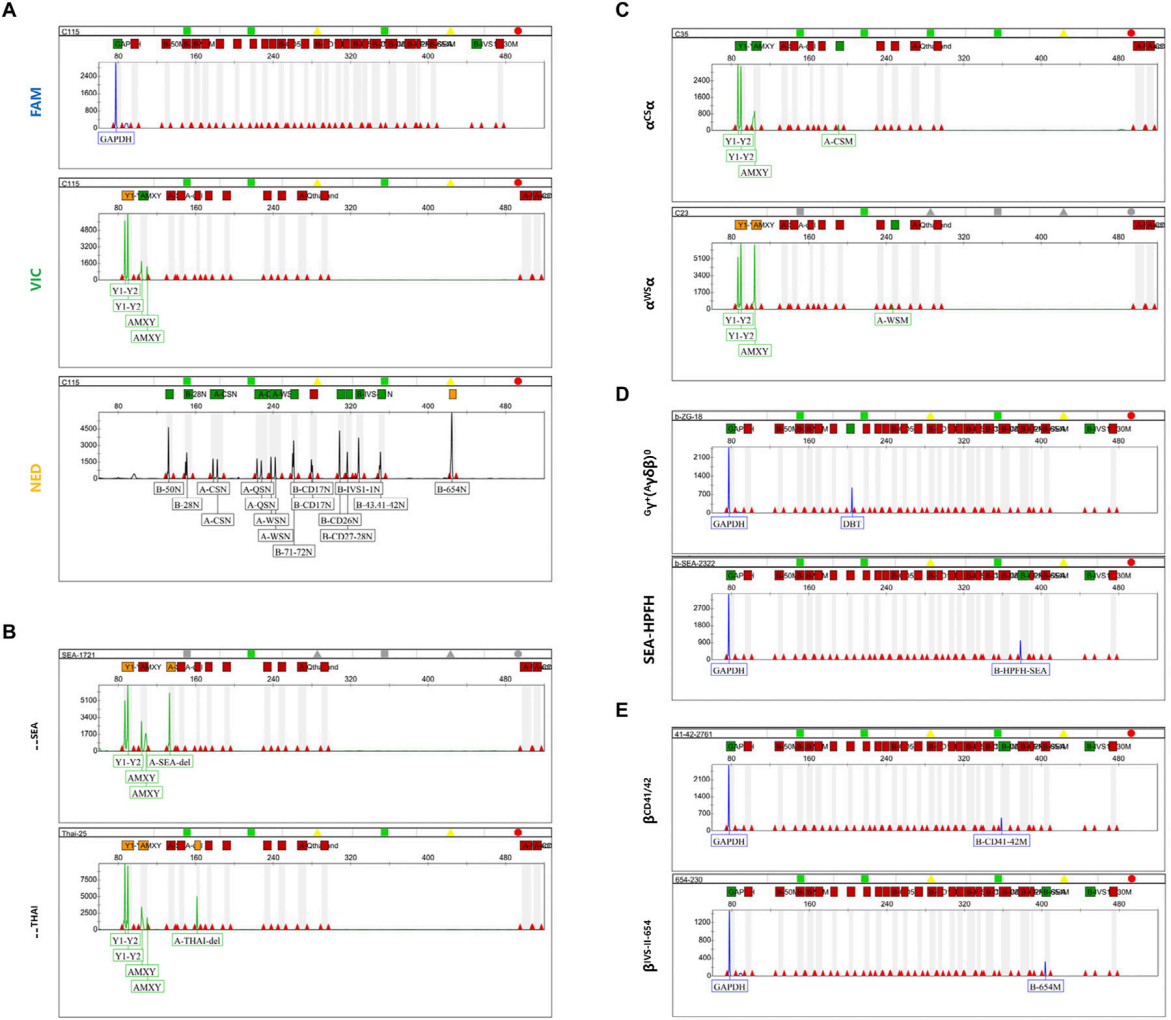
Capillary electrophoresis profiles of mutation peaks in different fluorescent channels. **(A)** The capillary electrophoresis profile of the wild-type αα/αα in three fluorescent channels. The GAPDH peak is in the FAM channel, the AMEL peak, Y1 and Y2 peaks are in the VIC channel, and the β-chain wild-type peak, α1 and α2 peaks are in the NED channel. **(B)** Capillary electrophoresis profile of the VIC channel for α-thalassemia deletion types. The upper represents the--^SEA^ type, while the lower represents the --^THAI^ type. **(C)** Capillary electrophoresis profile of the VIC channel for non-deletional α-thalassemia. The upper represents the α^CS^α type, while the lower represents the α^WS^α type. **(D)** Capillary electrophoresis profile of the VIC channel for β-thalassemia deletion types. The upper represents the Chinese ^G^γ^+^(^A^γδβ)^0^ type, while the lower represents the SEA-HPFH type. **(E)** Capillary electrophoresis profile of the VIC channel for non-deletional β-thalassemia. The upper represents the β^CD 41/42^ type, while the lower represents the β^IVS II−654^ type.

In addition to genotyping through ratio calculations, other thalassemia genotypes can be identified by mutation peaks in distinct fluorescent channels. After several rounds of primer design and screening, we conducted multiplex PCR amplification using two reaction tubes. Following capillary electrophoresis fragment analysis, the results showed that the housekeeping genes GAPDH in the FAM channel and AMEL in the VIC channel were successfully amplified in all samples (Figure 3A). No mutation peaks were detected at other sites in the FAM and VIC channels, and the wild-type peaks of the β-globin chain in the NED channel were all identified. In combination with the peak height ratio results being 1:1, the sample can be diagnosed as wild-type (Figure 3A). To evaluate the specificity of mPCR-CEFA, we analyzed 35 thalassemia genotypes other than those used for quantitative analysis. The results demonstrated that mutation peaks of various genotypes were distinguishable in their respective fluorescent channels, and genotypes could be accurately identified based on the size of the amplified products. Specifically, α-thalassemia variants were detected in the VIC channel (Figures 3B, C; Supplementary Figure S1), and β-thalassemia variants were detected in the FAM channel (Figures 3D, E; Supplementary Figure S2), with no cross-reactivity observed. For those non-deletion β-thalassemia genotypes designed with wild-type controls, if both mutation peaks and wild-type peaks are present, the sample is considered to be a heterozygote. If a mutation peak is detected in the FAM channel but the corresponding wild-type peak is absent in the NED channel, the sample can be diagnosed as a homozygote. The mPCR-CEFA method developed here allows the simultaneous detection of multiple genotypes across various samples, significantly improving detection efficiency.

### 3.3 Validation of the established method with clinical samples

We evaluated the performance of the method developed in this study by testing 200 clinical samples, all of which had been previously genotyped and confirmed. All method development and validation experiments were conducted within a single laboratory to ensure consistency in experimental procedures and minimize technical variability. These samples represented 40 distinct genotypes, including 15 negative and 185 positive cases. Using the mPCR-CEFA method, 15 negative samples were correctly identified (negative concordance rate of 100%) and 184 positive samples were correctly genotyped (positive concordance rate of 99.5%). One sample could not be interpreted. The overall detection accuracy of this method was 99.5% (Supplementary Table S3). Further analysis of the uninterpreted sample revealed that although the DNA concentration was within the acceptable range, sample degradation led to low DNA quality, resulting in capillary electrophoresis peak heights that failed to meet the analytical threshold. After re-extracting the DNA, the sample was successfully analyzed, yielding results consistent with the reference genotype.

**FIGURE 4:**
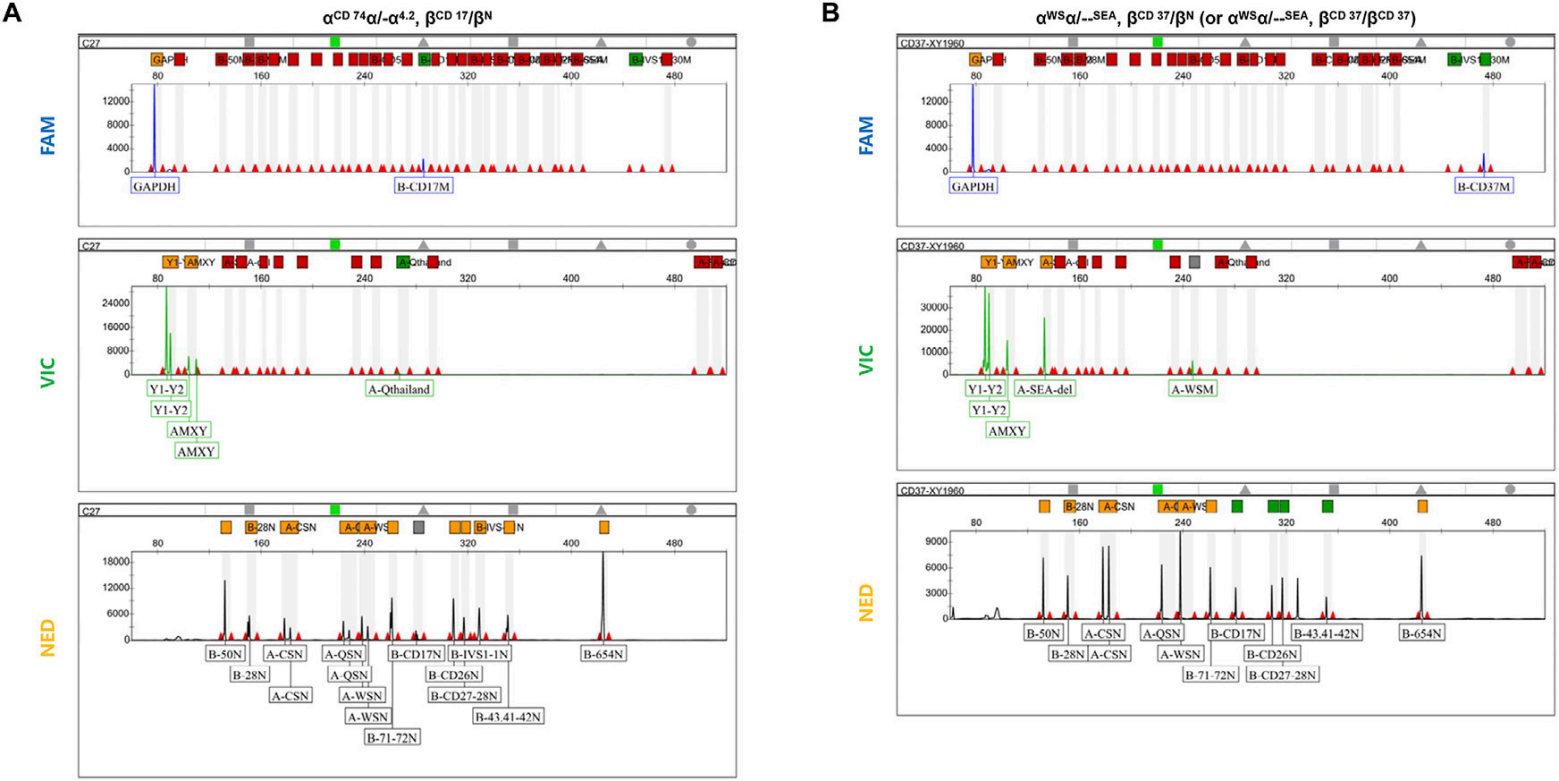
Detection of complex compound genotypes using mPCR-CEFA. **(A)** Capillary electrophoresis profile of the three fluorescent channels for the complex compound genotypes α^CD 74^α/-α^4.2^, β^CD 17^/β^N^. The α1/α2 peak height ratio is 2:1, and the Y1/Y2 peak height ratio is 2:1. The α^CD 74^α mutation peak is in the VIC channel. The β^CD 17^ mutation peak is in the FAM channel, while the β^CD 17^ wild-type peak is in the NED channel. **(B)** Capillary electrophoresis profile of the three fluorescent channels for the complex compound genotypes α^WS^α/--^SEA^, β^CD 37^/β^N^ (or α^WS^α/--^SEA^, β^CD 37^/β^CD 37^). The α1/α2 peak height ratio is 1:1, and the Y1/Y2 peak height ratio is 1:1. The α^WS^α and --^SEA^ mutation peaks are in the VIC channel, and there is no α^WS^α wild-type peak in the NED channel. The β^CD 37^ mutation peak is in the FAM channel, and its wild-type peak is not detected, making it impossible to distinguish whether the β^CD 37^ genotype is heterozygous or homozygous.

Furthermore, 175 clinical samples, representing 28 genotypes within the detection range of conventional thalassemia detection kits, were tested in parallel for comparison. The results showed that the mPCR-CEFA method achieved 100% concordance with the detection kits (Table 2), further validating its accuracy and reliability. Notably, the mPCR-CEFA detected 12 genotypes not covered by conventional detection kits and also successfully identified two cases of complex compound genotypes, including α^CD 74^α/-α^4.2^, β^CD 17^/β^N^ and α^WS^α/--^SEA^, β^CD 37^/β^N^ (or α^WS^α/--^SEA^, β^CD 37^/β^CD 37^) (Figures 4A, B). In these cases, single detection kits or genotyping methods alone struggle to detect such complex genotypes, often producing ambiguous or incomplete results.

**TABLE 2:** Comparison of diagnostic accuracy between mPCR-CEFA and conventional detection kits.

Variants	Numbers	mPCR-CEFA	Conventional technology
αα	15	15	15
-α^3.7^	24	24	24
-α^4.2^	20	20	20
--^SEA^	23	23	23
--^THAI^	5	5	5
-α^27.6^	3	3	3
α^CS^α	5	5	5
α^QS^α	5	5	5
α^WS^α	5	5	5
^G^γ^+^ (^A^γδβ)^0^	3	3	3
SEA-HPFH	3	3	3
β^−29^	4	4	4
β^−28^	3	3	3
β^Cap+40–43^	3	3	3
β^Init CD^	4	4	4
β^CD14/15^	2	2	2
β^CD17^	3	3	3
β^CD26^	4	4	4
β^CD27/28^	4	4	4
β^IVS I−1^	3	3	3
β^IVS I−5^	1	1	1
β^CD41/42^	5	5	5
β^CD43^	3	3	3
β^CD71/72^	4	4	4
β^IVS II−654^	3	3	3
ααα^anti−3.7^	5	5	5
ααα^anti−4.2^	4	4	4
Hkαα	6	6	6
fusion	3	3	3
Total	175	175	175

In terms of detection time, PCR-RDB typically requires 6–8 h due to its complex hybridization steps, while Gap-PCR is operationally simpler and requires less time about 5–6 h. In contrast, mPCR-CEFA combines multiplex PCR with automated capillary electrophoresis and completes the entire detection process within approximately 4–6 h. Taken together, these findings highlight the superior performance and practical utility of mPCR-CEFA in thalassemia detection.

## 4 Discussion

Thalassemia is a common hereditary blood disorder with a high prevalence in regions such as the Mediterranean, Africa, the Middle East, and South Asia, with China being among the most affected areas globally (Zhang et al., 2024). Accurate genetic diagnosis is crucial for the prevention and management of thalassemia. However, current diagnostic approaches face several challenges. Traditional methods, such as Gap-PCR and PCR-RDB, are widely used for detecting common variants but have limitations in simultaneously identifying multiple variants, interpreting complex genotypes, and screening for rare variants. Furthermore, these methods typically require extended detection times, limiting their suitability for rapid, accurate, and high-throughput screening (Luo et al., 2022a).

The mPCR-CEFA method developed in this study addresses these limitations by enabling the simultaneous detection of 16 α-thalassemia and 24 β-thalassemia variants in a single experiment, significantly expanding the detection scope and improving diagnostic efficiency. By employing capillary electrophoresis fragment analysis, the α1/α2 and Y1/Y2 peak height ratios were quantitatively calculated, and mutation peaks were distinguished and identified, ensuring high resolution and specificity in detection. The inclusion of internal quality controls allowed for comprehensive monitoring of the entire detection process, thus guaranteeing the validity of results. This is particularly critical for reducing the likelihood of misdiagnosis or missed diagnosis, especially in cases of silent or mild thalassemia. Moreover, compared to traditional methods that typically require multiple reaction setups and complex workflows, the two-tube reaction system and automated capillary electrophoresis of mPCR-CEFA simplify the procedure, reduce operational complexity, and significantly shorten detection time. This approach minimizes the risk of PCR product contamination in the laboratory while achieving high-throughput detection without sacrificing accuracy.

The method’s high concordance rate of 100% with conventional detection kits, and the overall accuracy of 99.5%, further highlights its reliability and robustness. The single case with an uninterpretable result underscores the importance of sample quality in diagnostic accuracy. These issues could be addressed by optimizing DNA extraction protocols or refining the analytical thresholds for capillary electrophoresis. Additionally, for complex compound genotypes, mPCR-CEFA successfully identified all samples, whereas conventional methods failed to provide definitive diagnoses for certain compound cases. Therefore, the mPCR-CEFA developed in this study serves as a valuable tool for large-scale screening and clinical applications.

In the context of public health, the application of mPCR-CEFA holds significant promise for the prevention and control of severe thalassemia. Early and accurate identification of carriers through genetic screening facilitates informed genetic counseling and prenatal decision-making, ultimately reducing the birth of children with severe thalassemia. This approach aligns with the thalassemia prevention strategies in China and other high-prevalence countries (Ip and So, 2013; He et al., 2017; An et al., 2024). Although the present study primarily focused on 40 variants in α- and β-globin genes, the principle and technical framework of mPCR-CEFA exhibits good scalability. However, the method has limitations, such as the lack of normal controls for certain clinically rare variant types, making it unable to distinguish between homozygous and heterozygous genotypes. If such variants are detected, further confirmation of genotypes using other methods, including Sanger sequencing and next-generation sequencing (NGS), is required. By optimizing primer design, this method can be further extended to cover more rare variants in thalassemia, as well as differentiate between heterozygous and homozygous genotypes for each type, providing possibilities for screening atypical thalassemia patients.

## Data Availability

The original contributions presented in the study are included in the article/Supplementary Material, further inquiries can be directed to the corresponding authors.
