# Supplementary material for: Development and validation of mPCR-CEFA for detecting multiple deletion and non-deletion thalassemia genotypes
Source: Front Genet. 2025 Jul 8;16:1564565. doi: 10.3389/fgene.2025.1564565 (PMC12282245; doi:10.3389/fgene.2025.1564565)
Supplement: Supplementary file 2 [file DataSheet1.docx]

Supplementary Material


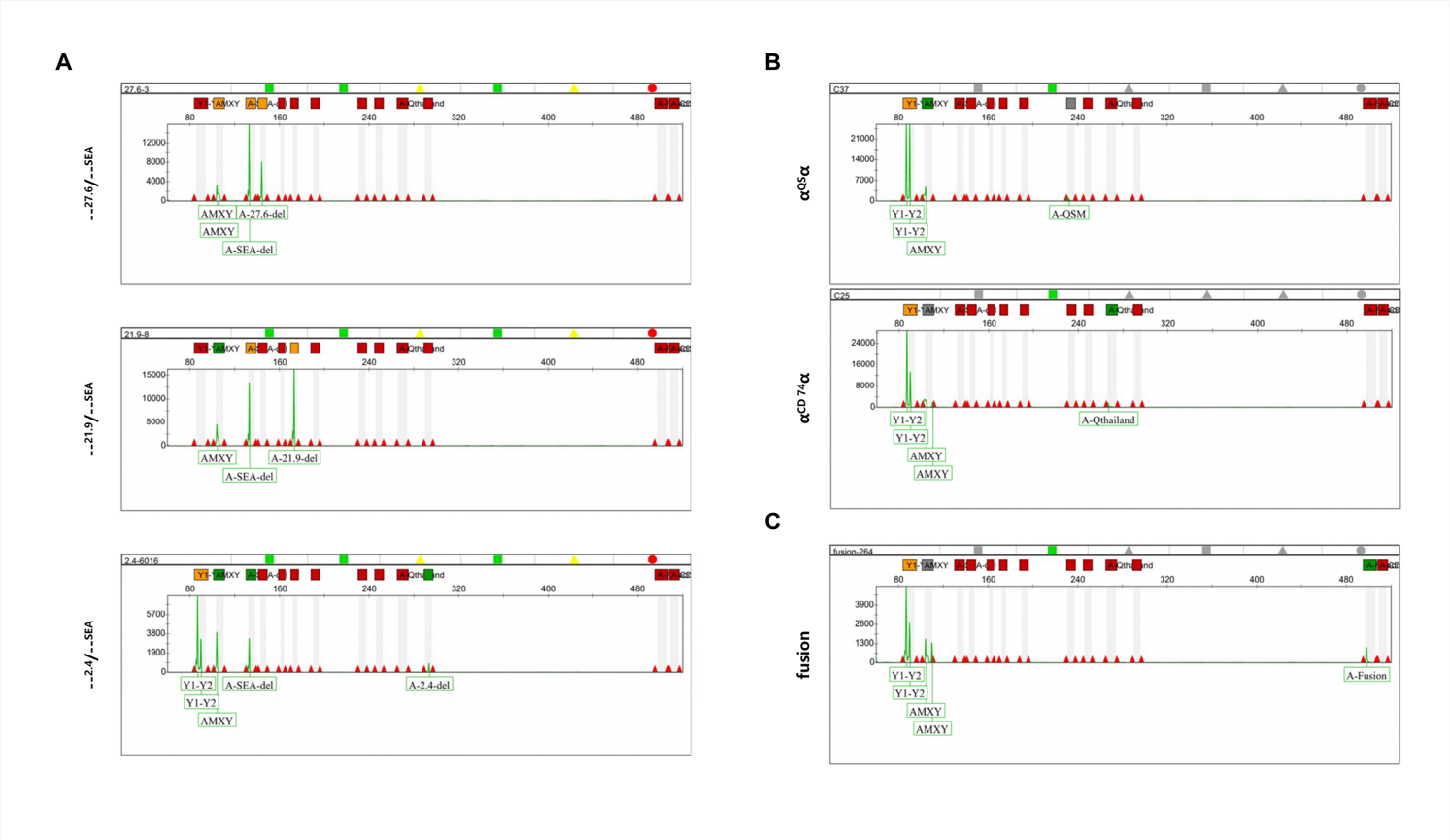


**Supplementary Figure S1. Capillary electrophoresis profiles of α-thalassemia genotypes.** **(A)** Capillary electrophoresis profiles of α-deletional thalassemia genotypes. From top to bottom: --^27.6^/--^SEA^, --^21.9^/--^SEA^, and --^2.4^/--^SEA^. **(B)** Capillary electrophoresis profiles of non-deletional α-thalassemia genotypes. The upper profile shows α^QS^α, and the lower profile shows α^CD 74^α. **(C)** Capillary electrophoresis profiles of α-thalassemia fusion gene.


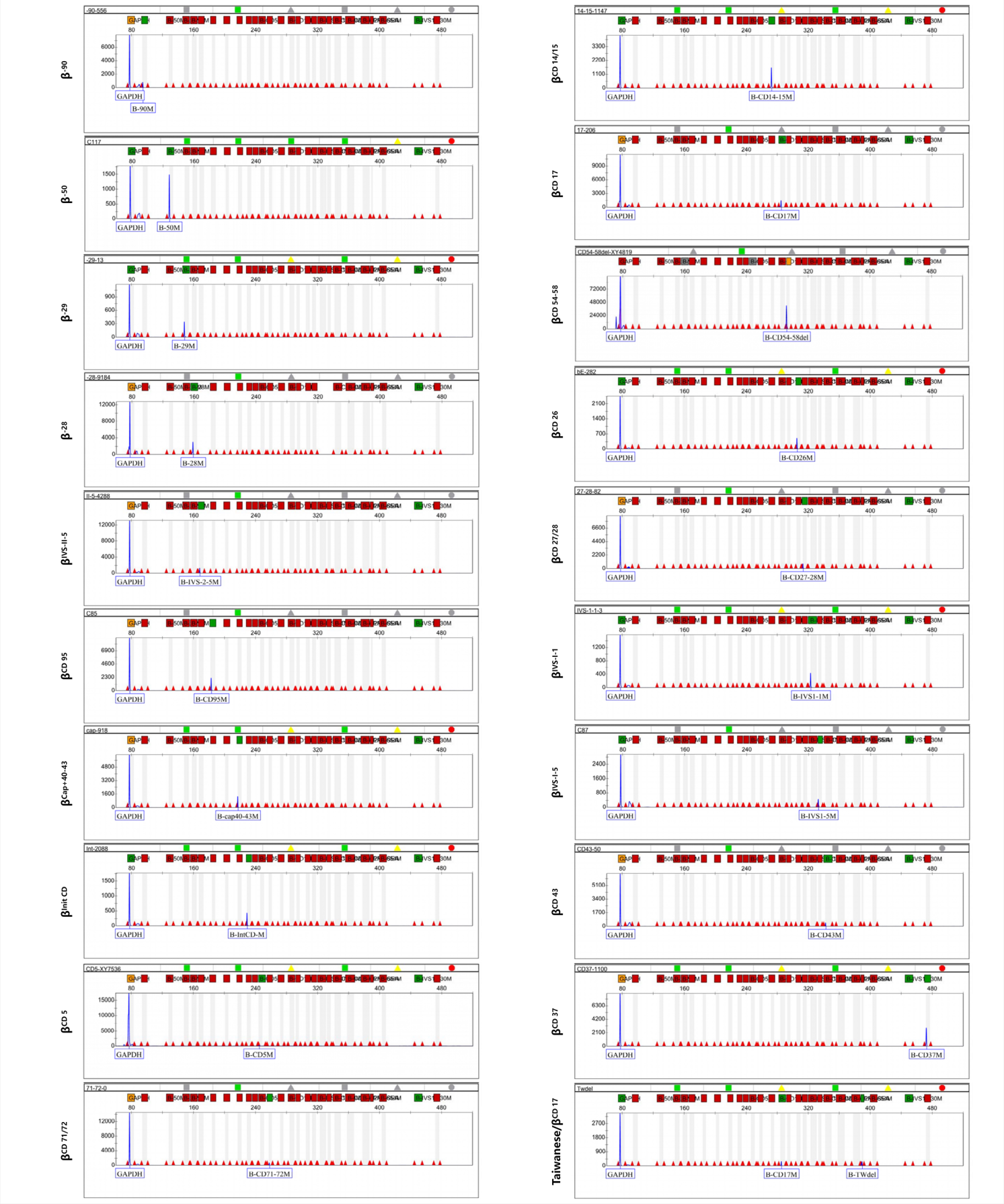


**Supplementary Figure S2.** **Capillary electrophoresis profiles of β-thalassemia genotypes.**
